# Clinical and Pathological Characteristics of Soft Tissue Sarcomas: A Retrospective Study From a Developing Country

**DOI:** 10.7759/cureus.9913

**Published:** 2020-08-21

**Authors:** Talal Almas, Muhammad Kashif Khan, Muhammad Faisal Murad, Muneeb Ullah, Adil Shafi, Maryam Ehtesham, Syed Muhammad Jawad Zaidi, Salman Hussain, Mehwish Kaneez

**Affiliations:** 1 Internal Medicine, Royal College of Surgeons in Ireland, Dublin, IRL; 2 Surgical Oncology, Federal Government Poly Clinic (Post Graduate Medical Institute), Islamabad, PAK; 3 Surgical Oncology, Maroof International Hospital, Islamabad, PAK; 4 General Surgery, Maroof International Hospital, Islamabad , PAK; 5 General Surgery, Maroof International Hospital, Islamabad, PAK; 6 Internal Medicine, Rawalpindi Medical University, Rawalpindi, PAK

**Keywords:** soft tissue sarcomas, clinical characteristics, management

## Abstract

Introduction

Soft tissue sarcomas remain an exceedingly rare malignancy. While soft tissue sarcomas boast a high mortality rate, their characteristics and behavior patterns are poorly understood. This study aims to evaluate the various aspects that pertain to soft tissue sarcomas, including their histology, tumor characteristics, survival rates, and therapeutic modalities.

Methods

A retrospective study analyzing the data from 19 patients presenting over four years with a histologically confirmed diagnosis of soft tissue sarcomas was conducted. The patients were studied for various parameters, including tumor site and the particular pathological subtypes. The data obtained were analyzed using the SPSS 23.0 statistical software (IBM Corporation, Armonk, NY), and the results were then tabulated.

Results

A total of 19 patients with a confirmed diagnosis of a soft tissue sarcoma were included in the study. The mean age of the patients included was 45.32 ± 16.88 years. Wide local excision was the most common surgical procedure employed for the resection of these tumors. Within the cohort, the mortality rate was noted to hover at 10.52%. Gastrointestinal stromal tumors were observed in 21% of the patients and were therefore the most common histological subtype. Of the patients included, 42.10% required blood transfusion during the perioperative time. Most of the tumors were noted to be intermediate grade, with high-grade tumors observed in 26.3% of the cases.

Conclusion

Soft tissue sarcomas remain a rare but potent cause of death in developing countries. The diversity of the tissues that they afflict renders their prompt detection a diagnostic challenge. A meticulous exploration of the various characteristics honed by soft tissue sarcomas, such as the particular histological subtype and the associated mortality rates, can better elucidate the prognosis and the eventual disease outcomes.

## Introduction

Soft tissue sarcomas (STS) refer to a rare group of heterogeneous tumors of mesenchymal origin and comprise less than 0.2% of all adult cancers [1]. Due to their predilection for evoking malignant transformations in a plethora of various tissues, STS are believed to be one of the most diverse malignancies [1,2]. In general, STS may involve the connective tissue of the head and neck, trunk, and limbs, as well as the retroperitoneum [2]. STS manifest a spectrum of tumor behaviour, ranging from indolent growth to widespread metastasis [3]. Pertinently, the optimal management of STS remains a conundrum for clinicians, and depends on a multitude of factors, including clinical characteristics, tumor characteristics, tumor size, and the histopathological subtype [2,4]. Due to the complexity of STS, a multimodal approach is usually followed, and the utilization of a multidisciplinary approach plays a vital role in the apt management of these tumors [5].

Retroperitoneal sarcomas (RPS) are a rare group of soft tissue malignant neoplasms that comprise merely 1%-2% of all solid cancers and only 10%-20% of all sarcomas [6-8]. These tumors classically arise in the retroperitoneum and have the potential to reach exorbitant proportions without eliciting any symptoms, and thus usually present late in the disease course. When RPS present with symptoms, they are usually non-specific and include abdominal pain, abdominal discomfort, fullness, and changes in urinary or bowel habits [6]. Complete surgical resection is the standard of care for RPS, with conflicting data on the use of adjuvant and neoadjuvant therapies [9-11]. The retroperitoneum contains multiple vital organs and critical structures, including the aorta, vena cava, head of the pancreas, and duodenum [2,6].

Sarcomas generally have a poor prognosis, with a five-year overall survival rate hovering around 36%-58% [6]. Overall survival is impacted by various prognostic factors that include histologic subtype, grade, and completeness of tumor resection [12]. While these are established prognosticators, additional parameters, such as tumor size, transfusion requirements, and anatomical location of the tumor, are also conjectured to impact the overall survival. Since surgical resection is the mainstay of management, different patterns of resection, including the employment of complex compartmental resection, are often required and are associated with varying complication rates and postoperative outcomes [13,14]. The goal is to achieve complete resection with negative macroscopic and microscopic margins to reduce the risk of local recurrence [6]. Due to the paucity of data elucidating the outcomes of STS in developing nations such as Pakistan, there is an unmet need to analyze these parameters as they pertain to STS. The present study therefore aims to delineate these parameters.

## Materials and methods

A retrospective cross-sectional study was conducted in the department of Surgical Oncology, Maroof International Hospital, Islamabad, Pakistan. A total of 19 patients who underwent surgery for a myriad of sarcomas involving various sites from January 2016 till January 2020 were included in the study. The patients were studied for various parameters, including tumor site, histopathological subtype, tumor grade, and the type of intervention employed. Patient comorbidities and various other surgical outcomes were also evaluated. Thereafter, the distribution of the various sarcomas in these patients was tabulated. The data were then analyzed using the SPSS 23.0 software (IBM Corporation, Armonk, NY). 

## Results

In the present study involving 19 cases, the mean age of the study participants was 45.32 ± 16.88 years, with a range of 20 to 80 years. Table 1 delineates the characteristics of study participants based on their gender, marital status, and comorbidities. 

**Table 1:** Background characteristics of the study participants.

Parameter		Frequency	Percentage
Gender	Male	10	52.6%
	Female	9	47.4%
Marital status	Married	15	78.9%
	Unmarried	4	21.1%
Comorbidities	Hypertension	2	10.5%
	Diabetes mellitus	2	10.5%
	Ischemic heart disease	1	5.3%

Based on the clinical evaluation, baseline laboratory investigations, and radiological imaging, the initial diagnosis was made. A plethora of various surgical procedures were deemed apt based on the site implicated and the extent of the tumor. Wide local excision was the preferred modality of surgical intervention in 16 patients, while compartmental excision was performed in merely 3 patients. Table 2 further highlights the primary site of involvement, the closest margins, the grade of the tumor, and the type of procedure employed. 

**Table 2:** A tabulation of the various parameters studied with pertinence to soft tissue sarcomas.

Parameter		Frequency	Percentages
Primary site of tumor	Gastrointestinal tract	3	15.8%
	Abdominal/pelvic wall	3	15.8%
	Breast	2	10.5%
	Retroperitoneal	3	15.8%
	Limbs	3	15.8%
	Others	5	26.3%
Grade of tumor	Low	6	31.6%
	Intermediate	8	42.1%
	High	5	26.3%
Closest margin	1-10 mm	6	31.6%
	10-20 mm	5	26.3%
	Greater than 20 mm	5	26.3%
	Involved	3	15.8%
Surgical procedure	Wide local excision	16	84.2%
	Compartmental excision	3	15.8%

The particular histological subtypes of the sarcomas were also evaluated. Table 3 delineates the frequency of the various histopathological subtypes. 

**Table 3:** The frequency of the various histopathological subtypes of sarcomas.

Histopathological subtype	Frequency
Gastrointestinal stromal tumor	4
Undifferentiated pleomorphic sarcoma	2
Retroperitoneal and dedifferentiated liposarcoma	2
Retroperitoneal leiomyosarcoma	1
Fibrosarcoma	1
Carcinosarcoma of uterus	1
Solitary fibrous tumor (malignant)	1
Endometrial stromal sarcoma	1
Neurofibrosarcoma	1
Myxoid leiomyosarcoma	1
Malignant spindle cell sarcoma	1
Malignant phyllodes tumor	1
Malignant peripheral nerve sheath tumor	1
Recurrent dermatofibrosarcoma protuberans	1

Imperatively, merely 2 out of the 19 patients eventually died due to recurrent and persistent disease. The postoperative outcomes of the surgical interventions performed are detailed in Table 4. 

**Table 4:** The postoperative outcomes of patients operated for various sarcomas.

Parameter	Frequency	Percentage
Mortality	2	10.5%
Blood transfusion required	8	42.1%
Need for re-exploration	2	10.5%
Need for re-admission	2	10.5%
Need for chemotherapy	4	21%
Need for radiotherapy	4	21%
Median operating time (range)	120 (60-240) minutes	
Median hospital stay (range)	3 (1-6) days	

## Discussion

STS remain a rare but diverse malignancy, notably affecting a vast range of different tissues and organs [1]. Imaging modalities, such as MRI, remain pivotal in detecting soft tissue tumors. CT and standard radiographs are used to rule out other possible bone tumors or cystic lesions [3,4]. After the appropriate radiological assessment, the gold standard diagnostic investigation is the core needle or excisional biopsy [5]. Given that sarcomas are noted to elicit a multitude of non-specific symptoms, they are often detected incidentally upon physical examination or imaging [6]. Ascertainment of the particular histologic subtype is performed through the means of image-guided percutaneous core needle biopsy, preferably with a co-axial technique to minimize the risk of seeding [15].

Before the consideration of the optimal management plan for the patient, a thorough preoperative evaluation of factors such as the patient’s age and comorbidity status should be performed. The overarching goal, and the most efficacious treatment modality for soft tissue tumors in general and RPS in specific, is complete resection of the tumor with negative microscopic and macroscopic margins [6]. However, an accurate pathological assessment of microscopic margins is often onerous and imprecise due to the exorbitant proportions that STS can grow to. It is therefore more pragmatic to aim for a complete macroscopical resection [16]. In certain subtypes of RPS, extensive encasement of adjacent structures is noted, which often warrants an en bloc compartmental resection approach for the excision of the tumor along with the encased structures. This usually presents a formidable challenge to surgeons owing to the proximity of the tumor to various vital organs [17].

Oncological literature vouches for the notion that the grade of the tumor remains the most imperative prognostic factor [18]. Although all types of STS manifest a spectrum of behavior, ranging from indolent to malignant, certain sarcomas have a lower metastatic potential or less aggressive behavior than the other subtypes. Other prognosticators include tumor size, depth of invasion, and tumor location, with retroperitoneal tumors often boasting a worse prognosis [3,18]. Of note, completeness of tumor resection, tumor grade, and histologic subtype are all factors that are intricately linked to the overall survival [19]. Avancés et al. reported that a high histologic grade was associated with tumor recurrence and poor survival [20]. In our data, 40% of the tumors with a high histologic grade were associated with recurrence or death. In contrast, a retrospective study observed that a high histologic grade was not associated with recurrence or poor overall survival [8]. Hassan et al. demonstrated an association between histologic subtype and overall survival, particularly outlining forbidding overall survival rates and outcomes portended by leiomyosarcomas [16]. In concert with this notion, our study divuled only two patients who were diagnosed with leiomyosarcomas, both of whom demonstrated poor prognostic outcomes. Patients with liposarcomas and lower grade sarcomas were found to have an improved overall survival [15]. 

Previous studies have established that complete surgical resection is the cornerstone curative treatment for RPS [21]. To this end, Malinka et al. analyzed predictors of overall survival and disease-free survival and identified surgical resection margins as one of the most important predictors of disease-free survival [22]. In our study, all patients who received blood transfusions had undergone either pelvic or abdominal surgery. Furthermore, half of those who received blood transfusions did not develop recurrence, metastasis, or a poor survival outcome. On the other hand, 25% of the patients who received transfusions eventually developed a recurrence. Whether receiving a blood transfusion serves as a viable prognostic factor remains shrouded in uncertainty and at the epicenter of an extensive oncological dilemma. 

Although surgical excision remains the cornerstone of STS management, a combination of radiotherapy and/or chemotherapy remains controversial. Despite an improvement in local control, the use of chemotherapy shows no improvement in overall survival [23]. This is in accordance with a retrospective analysis that reported no beneficial effects on survival with the use of chemotherapy; however, postoperative adjuvant radiotherapy displayed beneficial effects on overall survival for truncal sarcomas [24]. This notion is in contrast to a recent analysis of the Nationwide Clinical Oncology Database (NCOD) by Nussbaum et al. that established ameliorated survival outcomes with the uptake of radiotherapy in addition to surgery when compared to surgery alone [25]. Although surgical excision remains pivotal for portending favorable outcomes, the particular efficacy of radiotherapy and chemotherapy in the optimal management of STS remains elusive. Furthermore, background patient characteristics should also be factored into decisions pertaining to the optimal treatment regimens. There is thus an overarching need for the curation of specific guidelines that can better inform the debate on the most effective modality of management in patients with STS. Larger studies with greater sample sizes are needed in order to better elucidate the outcomes and characteristics of STS, especially as they prevail in developing nations such as Pakistan. 

## Conclusions

STS are a rare but important cause of cancer-related mortality in developed and developing countries alike. While surgical excision remains the cornerstone treatment modality, there is an ongoing debate on the efficacy of radiotherapy and chemotherapy in thwarting the carcinogenesis of STS. Due to their non-specific symptoms, STS can evade prompt detection, often presenting as high-grade tumors that portend grave disease outcomes. A meticulous evaluation through the means of history, physical examination, and radiological imaging, followed by a core biopsy to assess the local extent of disease, therefore remains imperative. Additional studies with larger sample sizes are needed to better delineate the characteristics and behavior patterns of these tumors in order to understand the disease prognosis more comprehensively.
